# Neuropilin (NRPs) Related Pathological Conditions and Their Modulators

**DOI:** 10.3390/ijms23158402

**Published:** 2022-07-29

**Authors:** Matic Broz, Anja Kolarič, Marko Jukič, Urban Bren

**Affiliations:** 1 Laboratory of Physical Chemistry and Chemical Thermodynamics, Faculty of Chemistry and Chemical Engineering, University of Maribor, Smetanova ulica 17, SI-2000 Maribor, Slovenia; matic.broz@um.si (M.B.); anja.kolaric2@um.si (A.K.); marko.jukic@um.si (M.J.); 2 Institute of Environmental Protection and Sensors, Beloruska ulica 7, SI-2000 Maribor, Slovenia; 3 Faculty of Mathematics, Natural Sciences and Information Technologies, University of Primorska, Glagoljaška ulica 8, SI-6000 Koper, Slovenia

**Keywords:** neuropilins, computer-aided drug design, in silico drug design, receptor modulator design, peptidomimetics, small-molecule antagonists, cancer, COVID-19, neuropathic pain

## Abstract

Neuropilin 1 (NRP1) represents one of the two homologous neuropilins (NRP, splice variants of neuropilin 2 are the other) found in all vertebrates. It forms a transmembrane glycoprotein distributed in many human body tissues as a (co)receptor for a variety of different ligands. In addition to its physiological role, it is also associated with various pathological conditions. Recently, NRP1 has been discovered as a coreceptor for the SARS-CoV-2 viral entry, along with ACE2, and has thus become one of the COVID-19 research foci. However, in addition to COVID-19, the current review also summarises its other pathological roles and its involvement in clinical diseases like cancer and neuropathic pain. We also discuss the diversity of native NRP ligands and perform a joint analysis. Last but not least, we review the therapeutic roles of NRP1 and introduce a series of NRP1 modulators, which are typical peptidomimetics or other small molecule antagonists, to provide the medicinal chemistry community with a state-of-the-art overview of neuropilin modulator design and NRP1 druggability assessment.

## 1. Introduction

**Figure 1:**
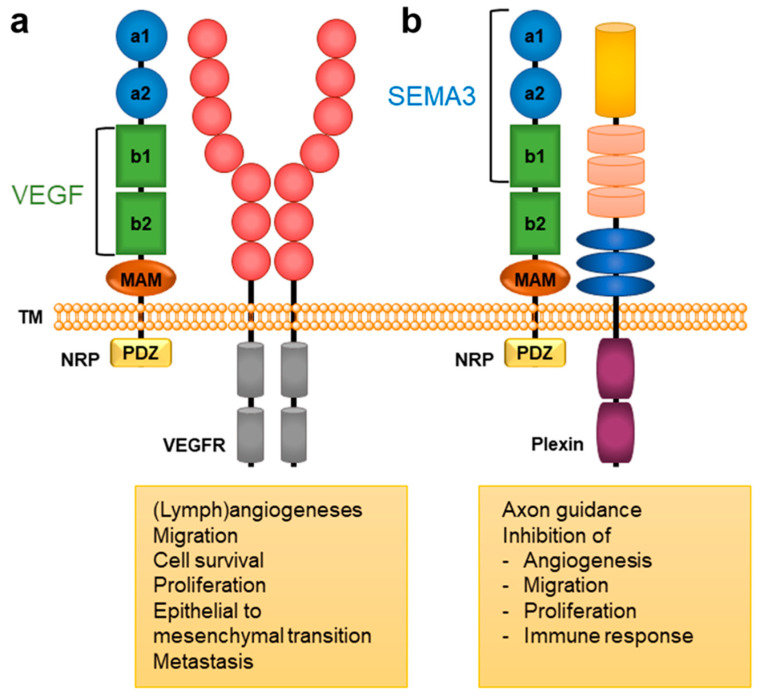
A general structural composition of NRP1 and NRP2 domains and the main NRP-mediated biological responses. The a1/a2 domain, presented in blue circles, is homologous to CUB (for complement C1r/C1s, Uegf, Bmp1); the b1/b2 domain, presented in green squares, is homologous to blood coagulation factor V/VIII domains; and the c domain, presented as an orange ellipse is homologous to meprin, A5, and μ-phosphatase (MAM). The intracellular PDZ domain is represented as a yellow square. Endogenous ligands of the VEGF family bind to the b1/b2 domains, while SEMA3s bind to the a1/a2/b1 domains [1,3]. (**a**) VEGFs form a complex with NRP and VEGFR that activates signalling pathways involved in angiogenesis associated with cancer [3,8]. (**b**) SEMA3s form a complex with NRP and plexin to activate signalling pathways that regulate axonal guidance and the immune, respiratory, and cardiovascular system as well as tumour cell responses [3,8,9].

Neuropilins (NRPs) represent transmembrane glycoprotein receptors important for the proper functioning of diverse biological processes due to their broad tissue distribution. They are mainly involved in neuronal development and axon guidance, angiogenesis [1], immune functions [2], and, consequently, also in the regulation of several pathological processes such as cancer, cardiovascular diseases [3,4], and viral infections [5]. NRPs lack direct signalling capabilities and act as coreceptors associating with other receptors to transduce a signal, primarily through various receptor tyrosine kinases [3]. There are two NRP types, NRP1 and NRP2, that share 44% sequence identity and exhibit a common domain structure. Their extracellular regions consist of 5 domains (Figure 1): a1/a2 domain, b1/b2 domain, and c (MAM) domain. The a and b domains bind particular endogenous ligands that trigger further signalling and provoke specific intracellular effects (Figure 1). In contrast, the MAM domain was initially thought to mediate NRP oligomerisation, but it more likely participates in the positioning of domains for their interactions with partner receptors by shielding them from the membrane [6]. The extracellular region is connected through a transmembrane (TM) domain to the short intracellular PSD-95/Dlg/ZO-1 (PDZ) binding domain, which lacks catalytic activity [1,3]. The mostly identical domain composition of NRP1 and NRP2 facilitates the involvement of both coreceptors in similar biological processes, yet they are still different enough to allow for distinct biological functions [7].

Extracellular domains of NRPs have defined, although not necessarily overlapping, binding sites that can accommodate various endogenous ligands and can interact with diverse receptors. NRPs are well known for their binding of class 3 semaphorins (SEMA3) [10,11] and selected members of the vascular endothelial growth factor (VEGF) family [12] that evoke different biological functions (Table 1). SEMA3s represent signalling proteins of a large and diverse semaphorins family, containing SEMA3A-3G subgroups that are involved not only in the guidance of axons and neural development [13] but also play important roles in immune, respiratory, and cardiovascular systems, as well as in pathological disorders, especially in tumour vasculature [3,8]. They bind with their C-terminal region to the a1/a2 and b1 domains of NRP [3], and since their binding is not sufficient for signal transduction, NRPs need to associate with the SEMA3 main receptor Plexin to form SEMA3-NRP-Plexin complex and to transduce the signal [14,15,16,17]. The members of the SEMA3 class exhibit different preferences for binding to NRP1 and NRP2 (Table 1), which results in various, more specific biological functions.

Vascular endothelial growth factor (VEGF) represents a family of signalling proteins involved in the development of blood vessels, including pathological angiogenesis as in cancer, vascular branching, and maturation, along with cardiovascular development [3,24]. The VEGF family consists of growth factors VEGF-A-D, as well as placenta growth factor (PlGF), parapoxvirus VEGF-E, and snake venom VEGF-F [25]. They primarily stimulate cellular responses by binding to their VEGF receptors (VEGFR). However, the binding of a VEGF to a coreceptor NRP forms a VEGF-NRP-VEGFR complex that results in enhanced VEGF signalling [24]. VEGFs bind to the b1/b2 domains of the NRP receptor, with the b1 domain being essential for the binding, while the b2 domain is required to ensure optimal binding [1]. The binding of VEGF ligands to b1 proceeds through the VEGF C-terminus sequence, containing a [R/K]XX[R/K] motif, called the C-end rule (CendR) [26]. Although the SEMA3 ligands also bind to the b1 domain, their binding site differs from one of the VEGF ligands [1]. As for SEMA3, there exists a distinct preference between NRP1 and NRP2 for different VEGF ligands (Table 1), which then perform specific endogenous tasks.

NRPs have also been identified as binding partners of other growth factors (Table 1), which demonstrates their versatility in regulating various signalling pathways. Thereby, NRPs can interact with the Fibroblast Growth Factor (FGF), the Hepatocyte Growth Factor (HGF), the Platelet-Derived Growth Factor (PDGF), the Transforming Growth Factor beta (TGF-β), and their respective receptors [3,9,21]. Moreover, NRPs have been reported to act as a receptor for extracellular microRNAs (miRNAs), which facilitates their internalisation into cells resulting in several physiological and pathological conditions. Thus, miRNAs have been associated with tumour progression, epithelial to mesenchymal transformation, metastasis and disease prognosis [2].

NRPs play an essential role in angiogenesis and lymphogenesis in endothelial cells through the binding of VEGF family members. The main pathway by which NRPs promote angiogenesis is through the formation of the NRP/VEGF/VEGFR complex, in which NRPs act as co-receptors with VEGFRs and enhance VEGF-induced activation of intracellular signaling pathways that consequently influence cell adhesion, migration, and permeability during angiogenesis under both physiological and pathological conditions [27,28,29]. NRP1 is mainly expressed in vascular endothelial tissue, whereas NRP2 is mainly expressed in lymphoid epithelium [30]. Although VEGF and its receptor, VEGFR govern angiogenesis, some studies have provided evidence that NRP1 and NRP2 can also promote blood vessel growth through alternative pathways [31,32].

Class 3 of NRPs endogenous ligands semaphorins also play an important role in vascular development, mainly by inhibiting angiogenesis. The semaphorins SEMA3A, SEMA3B, SEMA3D, SEMA3E, and SEMA3F interfere with VEGF-induced angiogenesis to promote their antiangiogenic effects [33].

Due to NRPs interacting with a broad range of endogenous ligands and triggering diverse physiological as well as pathological mechanisms, the modulation of their endogenous ligand binding exhibits a high potential for drug development. Therefore, small peptide ligands mimicking endogenous ligands have already been developed. Unfortunately, they lack metabolic stability and display a low bioavailability [24]. Moreover, the inhibition with monoclonal antibodies was also pursued, but significant side effects were observed [34]. Consequently, peptidomimetics, as well as small molecules, are gaining particular interest. Despite their limited size, they can interfere with the endogenous ligands binding to NRPs and have been reported to inhibit their signalling and biological functions [24]. Therefore, this article focuses on the latest findings on NRPs’ role in multiple diseases and attempts to review the NRP small molecule antagonists that could be used as successful therapeutic agents for the associated diseases.

## 2. NRP Binding of Endogenous Ligands

**Figure 2:**
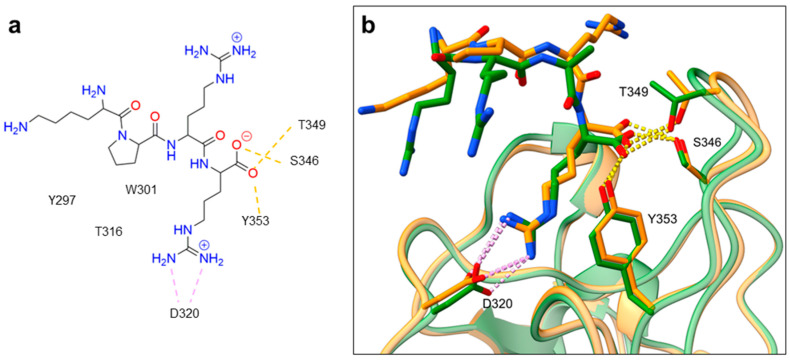
(**a**) A 2D representation of the VEGF-A165 CendR (KPRR) motif. Hydrogen bonding and salt bridge interactions, crucial for the high affinity of the VEGF-A165 terminal arginine with its NRP1 binding pocket, are depicted as yellow and light pink dots, respectively. (**b**) Superposition of NRP1 crystal structure complexes with VEGF-A165 (PDB ID: 4DEQ) [38] and SARS-CoV-2 (PDB ID: 7JJC) [37] CendR terminal residues bound into the NRP1 arginine binding pocket, yielding the comparison of the ligand-binding modes. VEGF-A165-NRP-1 complex is presented in orange cartoon and SARS-CoV-2 CendR-NRP-1 complex in green cartoon. NRP1 amino acid residues, significant for forming hydrogen bonds with the terminal arginine, are depicted as sticks of corresponding colours. Hydrogen bonding and salt bridge interactions are shown as yellow and light pink dots, respectively.

Despite NRP2 being an equivalently important target as NRP1, the latter has been more studied and better characterised. Although NRPs bind a large set of diverse endogenous ligands, little is known about the details of individual ligand interactions with its binding domain on the NRP receptor. The most studied and explored is the binding of VEGF-A165 to the b1 domain of NRP1, which serves as a basis for developing NRP small molecule antagonists (Figure 2). Due to the high structural similarity of both receptors, some of the NRP1 antagonists are able to extend their inhibitory activity on the NRP2-related biological signalling and functions. VEGF-A165 binds to the b1 domain of NRP1 with the C-terminal CendR motif, which has a terminal arginine residue. CendR facilitates the binding into a highly conserved b1 arginine binding pocket, consisting of amino acid residues Tyr297, Trp301, Thr316, Asp320, Ser346, Thr349, Tyr353 and Trp411 that were all recognised in a mutational analysis as crucial for a high VEGF-A165 affinity. The guanidine group forms a salt bridge with Asp320, and the free carboxylate interacts through hydrogen bonds with Ser346, Thr349, and Tyr353. Tyr297 and Tyr353 also participate in cation-π interactions with the CendR arginine side chains (Figure 2) [35]. In an additional exploration of the binding site region with synthetic ligands mimicking the terminal arginine, a hydration profile was analysed, thus revealing a conserved water molecule identified as important for increasing the ligand affinity by forming a hydrogen bond network between Trp301, Glu348, and the ligands [36].

The fact that the protein ligands binding to the b1 domain of NRP1 share a common C-terminal arginine motif is also evident from the recently solved crystal structure of SARS-CoV-2 CendR bound to NRP-1 [37]. The comparison of VEGF-A165 and SARS-CoV-2 CendR revealed almost identical binding modes, which share the interactions with the same key amino acid residues Tyr297, Trp301, Thr316, Asp320, Ser346, Thr349 and Tyr353 as depicted in Figure 2b. These residues seem to contribute to the binding affinity of all CendR-containing ligands; therefore, an interruption of interactions with these residues is deemed an attractive therapeutic approach.

## 3. Neuropilin-Related Pathology

### 3.1. Pain

Endogenous VEGF family ligands have been found instrumental in the pathophysiology of chronic pain in several diseases, including cancer, neuropathic pain, osteoarthritis, rheumatoid arthritis, migraine, and diabetic complications. Although mainly involved in the development and maintenance of pain, the best-studied VEGF-A165 shows not only pro- but also anti-nociceptive effects leading to analgesia [39,40]. These effects are due to the binding of different VEGF-A165 isoforms to their VEGFR receptors. The VEGF-A165a isoform is pronociceptive and leads to pain, while VEGF-A165b is considered anti-nociceptive, and the ratio between the two isoforms represents the key factor determining the effect on sensory neurons and pain levels [40,41]. VEGF-A binds to VEGFR and the coreceptor NRP1, which associates NRP1 with pain. While VEGF-A165a binds to VEGFR and NRP1 receptors, VEGF-A165b does not contain a CendR motif and, therefore, cannot bind to the NRP1 b1 domain [39,40]. Consequently, it appears that NRP1 is only involved in the activation of the pain pathway, making the VEGF-A165a/NRP1 complex an interesting target for reducing chronic pain.

### 3.2. Viral Entry

NRP receptors have been found to contribute to the infectivity of many viruses by enhancing their host cell entry (Table 2). This mainly involves viruses that contain the CendR motif, through which they bind to the b1 domain of the NRP1 receptor and this promotes the host cell infection. Among them, Epstein–Barr (EBV) [42] and Human T-cell lymphotropic virus type 1 (HTLV-1) [43,44] represent the best-studied ones. Recently, it was identified that the SARS-CoV-2 virus, the causative agent of the latest COVID-19 pandemic, also contains the CendR motif, which proposed NRP1 as its additional entry point into human cells [37,45].

Human T-cell lymphotropic virus type 1 (HTLV-1) represents the main cause of T-cell lymphoma and leukaemia. Cell-to-cell contact forms the major route of HTLV-1 infection, with NRP1 being essential for the viral entry. NRP1 is highly expressed in dendritic, T-cells, and endothelial cells, which represent the main targets of the HTLV-1 infection [43]. HTLV-1 enters host cells via a three-step process. First, the viral surface unit attaches to the heparin/heparan sulfate proteoglycan (HSPG) on the host cell surface. Then the HSPG/virus surface complex interacts with the b1 domain of NRP1, which triggers conformational changes of the viral surface that enable its interaction with glucose transporter GLUT-1, yielding HTLV-1 fusion and cell entry [50].

Epstein–Barr virus (EBV) represents human herpesvirus 4 (HVV4) implicated in malignancies of lymphoid or epithelial origin. Glycoprotein B (gB) in the virus envelope forms a critical factor for the infection of B and epithelial cells, whereby NRP1 was demonstrated as the main attachment point of gB, facilitating viral entry and the infection of nasopharyngeal epithelial cells, while its connection to B cells remains unknown. Upon EBV binding to nasopharyngeal epithelial cells, furin cleaves gB, exposing a CendR motif that binds to NRP1 and promotes viral fusion and internalisation into host cells. Moreover, the binding of EBV to NRP1 also activates receptor tyrosine kinase (RTK) signalling, which consequently promotes EBV infection of nasopharyngeal epithelial cells, but the detailed mechanism remains unknown [42]. With both viruses being highly involved in carcinogenic processes, it is assumed that they compete with endogenous VEGF ligands for the binding to NRP1 and might trigger cell signalling to promote the formation of tumour vessels in cancer tissues [51,52].

COVID-19 is primarily a disease of the respiratory system causing mild to severe respiratory symptoms, although expression in the central nervous system (CNS) has also been observed, implicating neurologic manifestations [53]. The entry of SARS-CoV-2 into host cells is mainly mediated by the cellular receptor angiotensin-converting enzyme 2 (ACE2) [54,55]. However, due to relatively low levels of ACE2 in pulmonary tissues, NRP1 was identified as an important partner for interacting with the virus and facilitating its entry [37,45]. To infect human cells, the SARS-CoV-2 spike protein is cleaved by the host furin protease at the S1/S2 junction [56], exposing a CendR motif, which binds to the b1 region of NRP1 [26] and provides viral fusion. Higher expression of NRP1 potentiates the infectivity with SARS-CoV-2 due to an increased viral entry rather than an enhanced binding [30]. NRP1 was also identified as a specific surface marker for T cells and is constitutively expressed on the surface of CD4 and CD25 cells and its expression is modulated depending the cell activation [57]. Furthermore, it was demonstrated that NRP1, is expressed at high levels on T regulatory cells and can be used to separate nT reg versus iT reg cells in certain physiological settings [58]. Namely, function of regulatory T cells is maintained by NRP1—semaphorin pathway where T cell function and survival is potentiated at inflammatory sites where is is especially important to limit anti-tumour immune responses [59,60]. As the regulatory T cells have a crucial role in the immune system by preventing autoimmunity, and maintaining immune homeostasis, their possible role and that of the surface NRP1 receptor in SARS-CoV-2 infection have been discussed [61]. Since COVID-19 represents a worldwide pandemic, there have been numerous reports of infected individuals that did not develop COVID-19 symptoms [62,63]. In some cases, the viral load of asymptomatic individuals has been equal to that of symptomatic individuals, hinting that the virus might interfere with neuropathic pain signalling pathways. It was found that the spike protein of SARS-CoV-2 competes with VEGF-A for the binding to NRP1 and inhibits VEGF-A signalling. This results in analgesia in asymptomatic COVID patients and might help spread the disease [64].

Some of the recent studies found that NRP1 may serve as an entry point also for other viruses, including β-herpesvirus murine cytomegalovirus (MCMV) [46] and Enterovirus 71 (EVA71) [47]. Further investigations are needed to elucidate its exact role and mechanism. Apart from NRP1, its homologous form NRP2 was found to contribute to the infection by certain viruses as well, namely, Lujo virus (LUJV) [48] and human cytomegalovirus (HCMV) [49] which use NRP2 as their viral entry point. NRPs could, therefore, form promising therapeutic targets for preventing viral infections and related diseases.

### 3.3. Cardiovascular Diseases

NRPs are involved in angiogenesis and cardiovascular diseases. On one hand, the knockout of NRP1 from cardiomyocytes and vascular smooth muscle cells causes cardiomyopathy, aggravated ischemia-induced heart failure, and hereditary haemorrhagic telangiectasia arteriovenous malformations, thus revealing its cardioprotective role [65,66]. On the other hand, NRP1 mediates the activation of human cardiac fibroblasts [67]. NRPs significantly contribute towards cardiovascular disease and the latter represent a serious comorbidity in COVID-19 patients [68,69,70,71,72].

### 3.4. Diabetes

The role of NRP in diabetes pathology was reviewed in 2002 by Mamluk et al. [73]. Especially after the outbreak of COVID-19 disease, NRP has been studied in more detail as a viral co-receptor and via involvement in co-morbidity [69]. Its involvement can be observed in diabetic nephropathy and the presence of NRP1 inhibitor proof-of-concept peptide compounds is of great interest [74,75]. However, the association between NRP1 and SARS-CoV-2 infection can be summarized in two of the most described scenarios [76,77].

Patients with diabetic nephropathy represent a group at higher risk for COVID-19 disease severity. NRP1 is found in the kidney, particularly in podocyte cells, where it is important for proper podocyte function, such as adhesion to extracellular matrix proteins, cytoskeletal reorganisation, and apoptosis. Its role is therefore important in diabetic nephropathy, in which it has been demonstrated that suppression of NRP1 expression may be responsible for podocyte damage and loss, leading to deterioration of renal function. It is speculated that the high expression of NRP1 in the kidney of diabetic patients facilitates the invasion of SARS-CoV-2 into this tissue, while the interaction of both processes leads to depletion of NRP1, which then exacerbates the pathogenesis of diabetic nephropathy. However, further research is needed to refine the current understanding of the potential role of NRP1 in diabetic nephropathy, particularly in conjunction with COVID-19 [78,79].

It was discovered that insulin-producing pancreatic β-cells express ACE2 and TMPRSS2 at low levels, whereas NRP1 expression is high in patients with COVID-19 [80,81]. Infection of pancreatic β cells with SARS-CoV-2 attenuates pancreatic insulin levels and secretion and induces β cell apoptosis, which was partially reduced by NRP1 inhibition. Therefore, it could be speculated that NRP1 supports viral infection in patients with type II diabetes [81].

### 3.5. Cancer

Cancer remains the second most common cause of death worldwide, responsible for almost 10 million deaths in 2020 alone [82]. While cancer, in most cases, takes years to develop into a life-threatening disease, it is usually discovered only after it has metastasised to other organs. Therefore, the metastatic potential of cancer cells remains one of the main prognostic factors for the overall survival of cancer patients. Over the last 5 years, more than 100 studies linking NRPs to various cancer types, mainly leukaemia, breast cancer, colon and colorectal cancer, lung, and liver cancer, have been published. NRP1 overexpression in cancer cells has been associated with tumour aggressiveness, enhanced cell proliferation, and metastasis. The literature on this topic is collected in Table 3 for the reader’s benefit.

Moreover, the other member of the neuropilin family, NRP2, also contributes to the cancer progression. For example, it was shown that NRP2 is expressed during macrophage differentiation, promotes efferocytosis, facilitates tumour growth [83] and promotes mobilisation [84]. In contrast, its deletion downregulates tumour-promoting genes, increases secondary necrosis within tumours and impairs apoptosis [83]. NRP2, but not NRP1, is expressed in cytokine-induced killer cells, which are responsible for the controlled apoptosis [85] of precancerous cells.

**Table 3:** Scientific literature collection on NRP1 and NRP2 involvement in different cancer types.

Cancer Type	NRP1	NRP2	Reference
Leukaemia	x		[86,87,88,89,90,91]
Breast cancer	x	x	[92,93,94,95,96,97,98,99,100,101,102]
Carcinoma	x	x	[103,104,105,106,107,108,109,110,111,112,113,114,115,116]
Colon & Colorectal cancer	x	x	[117,118,119,120,121,122,123,124,125,126]
Gastric cancer	x		[127,128,129,130,131,132,133,134,135,136,137,138]
Lung cancer	x	x	[139,140,141,142,143,144,145,146,147,148,149]
Pancreatic cancer	x		[150,151,152,153,154]
Prostate cancer	x	x	[155,156,157]
Melanoma	x	x	[158,159,160,161]
Glioma	x		[162,163,164,165,166,167,168]
Liver cancer	x		[169]
Mammary stem cells cancer	x		[170,171]
Esophageal cancer	x		[172,173]
Stem cell cancer	x		[174,175]
Thyroid cancer		x	[176,177]
Multiple myeloma	x		[51]
Lymphoma	x		[178]
Bladder cancer		x	[179,180]
Tongue cancer		x	[181]
Cervical cancer	x		[182,183]
Gallbladder cancer	x		[184]
Endometrium cancer	x	x	[185,186]

## 4. Neuropilin-1 Modulators

While peptide antagonists and monoclonal antibodies have failed to fulfil expectations [34], several NRP1 peptidomimetic as well as small molecule antagonists have been developed and were able to successfully inhibit the VEGF signalling. Interestingly, some of them (especially the small molecules) lack terminal arginine but were still able to achieve the desired effect. Therefore, peptidomimetic and nonpeptide small molecule drugs may represent an elegant and promising approach toward developing NRP1 antagonists. This review summarises the latest drug discovery findings accordingly.

### 4.1. Peptidomimetics

Peptidomimetic antagonists have been developed prior to peptide compounds to provide higher stability and drug-like properties (Table 4). The anticancer therapeutic strategy represents the largest part of NRP1 drug discovery efforts, aiming to prevent the VEGF binding and restrict the intracellular signalling. To that end, the peptidomimetic antagonists reported in the scientific literature were all developed for anticancer treatment and are imitating the CendR motif of VEGF ligands. The first NRP1 peptidomimetic, named EG00229 (compound **1** in Table 4), was based on mimicking the minimal peptide KPAR sequence of VEGF-A165 that could retain its activity on NRP1. The C-terminal arginine, which is supposedly crucial for this activity, was fully conserved and was connected through sulfonyl amino thiophene to benzothiadiazole heteroaryl, mimicking the lysine of KPAR. EG00229 inhibited the binding of VEGF-A to NRP1 with IC_50_ = 3 µM and was able to inhibit the VEGF-A mediated biological function partially [35]. Moreover, EG00229 was able to suppress glioma progression in mice [187]. The crystal structure of the EG00229-NRP1 complex confirmed its binding mode as similar to VEGF-A (Figure 2) with an identical C-terminal arginine position (Table 4). Furthermore, the complex revealed an intramolecular hydrogen bond between amide NH and sulfonamide nitrogen that provides the stability of the ligand conformation and is believed to be the reason for the overall biological activity [35]. Aiming to improve the H-bonding network, the benzothiadiazole heteroaryl moiety of EG00229 was replaced with methylaminoaryl-substituted dihydrobenzofuran, providing the compound EG01377 (compound **2** in Table 4) with an improved inhibition of VEGF-A binding to NRP1 with IC_50_ = 0.6 µM. Crystallographic studies revealed two crystal complexes in higher and lower resolutions, which differ in the binding conformation of the bulky aromatic moiety. The lower resolution structure was considered more correct and is presented in Table 4, whereby the amine of methylaminoary forms an H-bond with Glu348. Importantly, the compound exhibited good in vitro anti-angiogenic, anti-migratory and antitumour effects and, therefore, yields a high potential for further in vivo studies [188]. In a recent in silico study, the binding mechanism of EG00229, EG01377 and SARS-CoV-2 CendR was investigated, indicating that EG01377 indeed provides the strongest binding with the NRP1 b1 domain among all three studied ligands, and identified D320 as the key binding residue [188].

The idea of replacing the amino-acid residue of peptide antagonists with sugar-based fragments has emerged from yet another research group. Their peptidomimetics were prepared based on the ATWLPPR heptapeptide, an effective antagonist of the VEGF binding to NRP1 [189,190]. A rigid trioxabicyclo system mimics the LPP sequence linked with arginine-like functionalities to provide the required C-terminal, while tryptophane/threonine-like functionalities were introduced on the other end. The best binding compound **3** (Table 4) with IC_50_ = 92 μM did not provide the desired efficiency [191]; therefore, further optimisations were made on both residue-mimicking ends, exploring different structural motifs, varying their lengths and spatial orientations. These modifications led to the discovery of compound **4** (Table 4) with an improved inhibition of the VEGF-A binding to NRP1 with IC_50_ = 39 μM [192].

**Table 4:** Peptidomimetic NRP1 antagonists.

Compound	Binding Mode	Inhibition of VEGF-A Binding [μM]	Ref.
Cancer			
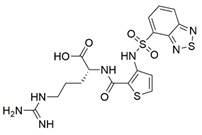 **1** (EG00229)	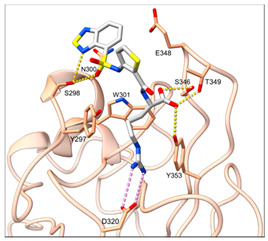 ^[a]^	IC_50_ = 3	[35]
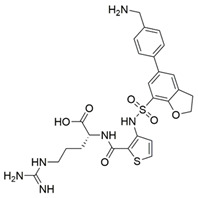 **2** (EG01337)	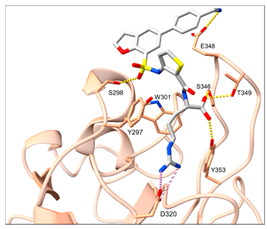 ^[b]^	IC_50_ = 0.6	[188]
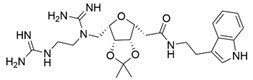 **3**	NA	IC_50_ = 92	[191]
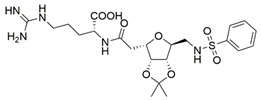 **4**	NA	IC_50_ = 39	[192]

[a] The crystal structure of EG00229-NRP1 complex (PDB ID: 3I97) [28]. [b] The crystal structure of EG01337-NRP1 complex (PDB ID: 6FMF) [160]. NRP-1 is depicted in orange cartoon representation, while ligand is in grey stick representation. NRP1 key amino acid residues involved in interactions with the ligand are shown as sticks in orange colour. Hydrogen bonding and salt bridge interactions are presented as yellow and light pink dots, respectively.

### 4.2. Small-Molecule Antagonists

The development of small molecule NRP antagonists is known to be challenging due to a large and flat protein-protein binding interface. Still, some fully non-peptidic small-molecule NRP1 antagonists have been successfully developed and exhibited potency in inhibiting the NRP1 mediated biological functions. The main focus of small molecule development was primarily on achieving anticancer activity. Nowadays, the knowledge of NRPs’ involvement in several diseases has expanded the development and studying of antagonists to new areas to provide novel therapeutic agents. Accordingly, the current focus is directed toward searching for the desired and necessary small-molecule medicines for the treatment of SARS-CoV-2 infections. The important small molecule antagonists are collected in Table 5.

In 2014, the first fully non-peptidic VEGF-NRP antagonist was identified in a virtual screening procedure. This hit compound **5** (Table 5), named NRPa-47, showed anti-angiogenic and antitumour effects in in vitro and in vivo assays and inhibited both NRP1 and NRP2, with selectivity over proangiogenic receptors. According to molecular modelling studies, its benzimidazole fragment was predicted to replace the C-terminal arginine of VEGF-A and peptide/peptidomimetic antagonists. Thus, this compound represents an auspicious starting point for the further development of cancer treatment medicine [193]. Pursuing ambitious research and development of NRP small molecule antagonists, the same research group identified yet another set of compounds with a common original molecular scaffold, whereby compound NRPa-308 (compound **6** in Table 5) emerged as the most promising new hit. NRPa-308, which structurally differs from NRPa-47, demonstrated remarkable anti-angiogenic and anti-proliferative effects in vitro. It was able to highly reduce (by more than 60%) the tumour growth of human breast cancer cell lines MDA-MB-231 and BT549 [194,195]. The binding mode elucidated by molecular docking calculations suggested that NRPa-308 forms H-bonds with Glu348 and Trp301 while its aromatic ring inserted deeply in the NRP1 arginine binding pocket is stacked between Tyr297 and Tyr353 displaying potential hydrophobic and/or aromatic interactions (Table 5). Interestingly, the salt-bridge interaction with Asp320 was not identified as a crucial element for the binding of antagonists [194]. Altogether, NRPa-308 represents one of the most promising anticancer compounds targeting NRP1.

In the same year, another research group discovered several antagonists that can inhibit VEGF-A binding to NRP1 in in vitro assays. These antagonists share a common chlorobenzyloxy alkyloxy halogenobenzyl amine scaffold (compound **7** in Table 5). According to molecular docking calculations, the scaffold is involved in common interactions such as H-bonds of the NH group with the OH group of Thr316, two-cycle substituted oxygen atoms with the NH group of Trp301 and carboxylic group of Glu348. Stacking interactions were observed with Trp301 and hydrophobic with Tyr353. Again, the salt bridge with Asp320 was not observed among the compounds exerting antagonistic effects and sharing a common scaffold; therefore, it seems this interaction should not be crucial for containing a proper anticancer activity [196].

Yet another series of non-peptidic small-molecule NRP1 antagonists emerged from a structure-based virtual screening, whereby compound **8** in Table 5 exhibited the strongest activity with IC_50_ of 19.1 µM. Interesting about this compound is its binding mode, which was elucidated by molecular dynamics simulations, and revealed the stacking of both aromatic rings with three stable π-π interactions formed with Tyr297, Trp301 and Tyr353 (Table 5). The aromatic rings achieve a much higher occupancy of the arginine binding pocket compared to arginine-based fragments. Such a binding mode is most likely responsible for the observed inhibitory activity [197].

Since it was discovered that VEGF-A/NRP1 signalling is also involved in neuropathic pain behaviour, it became an interesting novel target for the analgesic treatment. With the intention to find small molecule inhibitors that could interfere with the VEGF-A/NRP1 neuropathic pain signalling, a virtual screening protocol was developed, yielding several compounds with potential analgesic effects (compounds **9** and **10** in Table 5) [64]. As the newest discoveries emphasised the involvement of NRP1 also in SARS-CoV-2 infections [37,45], the hit compounds were further assayed for the SARS-CoV-2 spike-dependent antiviral activity. Compounds **10** and **11** (Table 5) displayed more than 50% inhibition of the viral activity, which represents the first step toward the development of small molecules with the potential of inhibiting the SARS-CoV-2 viral entry. The research group also proposed a pharmacophore model, which should maximise the binding pocket occupancy and contacts to facilitate the further design of NRP1 small molecule antagonists [40]. This research is among the first to consider the effect of hit compounds on multiple diseases, which represents a vital step toward developing small-molecule inhibitors of the VEGF-A/NRP1 signalling for the treatment of neuropathic pain, cancer, and potentially also of SARS-CoV-2 infections.

In an accelerated search for small molecule drugs active against SARS-CoV-2 infections, fast and less costly in silico studies have disclosed a promising starting material for further development. In addition to new chemicals, natural and FDA-approved drugs were also investigated against NRP1 from the Egyption sequence in a molecular docking study, which revealed that Hesperidine, Ravidasvir, Daclatasvir, Remdesivir, and Sofosbuvir presumably form favourable interactions with NRP1 accompanied by the lowest binding energy. This has raised the potential for drug-like natural products and existing drugs as future NRP1 inhibitors [198]. Another study emerged by screening the accessible library of drug-like small molecules and calculating their binding free energy, which in the end gave 10 compounds with a presumably more specific and stronger binding compared to the known NRP1 inhibitors EG00229 and EG01377 [199]. In the latest in silico study molecular compounds previously investigated in COVID-19 related studies were screened against NRP1 to obtain that Nafamostat, Y96, Selinexor, Ebastine and UGS may emerge as good candidates for preventing the binding of spike to NRP1 [200]. A growing interest and related research results point toward the opportunity to develop efficient and selective drugs against pathologies involving the NRP1 signalling.

Recently, our research group identified two additional compounds, **12** and **13** (Table 5), in a spike-NRP1 binding assay that exhibited a stronger inhibition of the spike CendR binding to NRP1 than the well-known NRP1 antagonist EG00229 (**1**). Both compounds were able to inhibit more than 60% of the spike binding, while EG00229 inhibited only 50% of binding, suggesting that these compounds represent a good starting point for the development of small molecule SARS-CoV-2 antagonists. The binding mode of both compounds within the CendR binding pocket of NRP1 was predicted by molecular docking, which revealed that both compounds presumably form strong hydrogen-bonding and salt bridge interactions with key amino-acid residues Asp320, Ser346, Thr349, and Tyr353, as shown in Table 5. According to the predicted binding modes, the binding pocket is not fully occupied, leaving room for a further optimisation of compounds that could enhance the binding and potentially lead to stronger antagonistic effects [201].

**Table 5:** Small-molecule NRP1 antagonists.

Compound	Binding Mode Prediction	Inhibition of VEGF-A Binding [μM]	Ref.
Cancer			
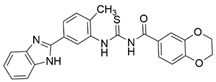 **5** (NRPa-47)	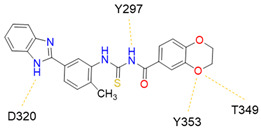	IC_50_ = 34	[193]
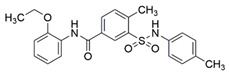 **6** (NRPa-308)	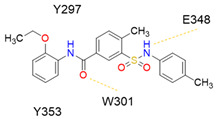	32% ^[a]^	[194]
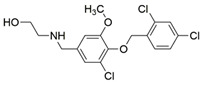 **7**	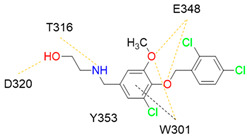	Ki = 7.3	[196]
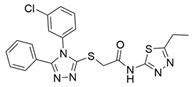 **8**	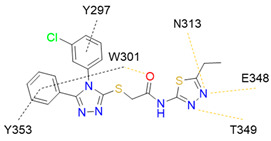	IC_50_ = 19.1	[197]
Neuropathic pain			
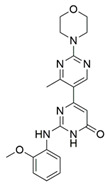 **9**	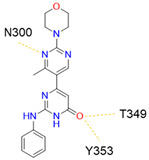	IC_50_ = 0.000598	[40]
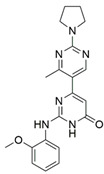 **10**	NA	IC_50_ = 0.00314	[40]
COVID-19		SARS-CoV-2 inhibition [%]	
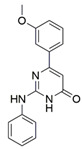 **11**	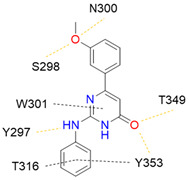	>50 ^[b]^	[40]
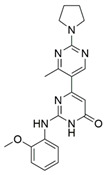 **10**	NA	>50 ^[b]^	[40]
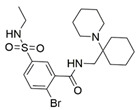 **12**	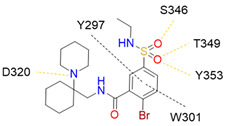	61.44 ± 2.48 ^[c]^	[200]
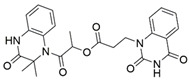 **13**	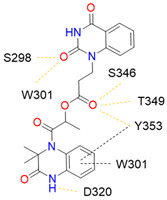	63.58 ± 1.27 ^[c]^	[200]

^[a]^ VEGF-A165/NRP-1 binding inhibition at 10 μM compound concentration. ^[b]^ SARS-CoV-2 entry inhibition at 25 μM compound concentration. ^[c]^ SARS-CoV-2 spike S1 binding inhibition to NRP1 at 100 μM compound concentration. Hydrogen bonding and π-π stacking interactions are presented as yellow and black dots, respectively.

**Figure 3:**
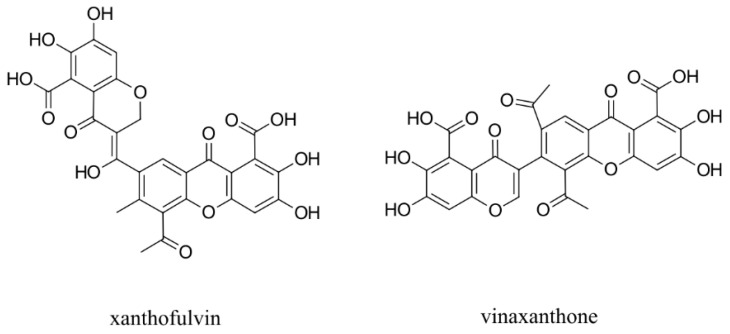
Natural compound SEMA3A inhibitors.

Regarding semaphorins, attempts have been made to interfere with the SEMA3/NRP binding. Natural compounds xanthofulvin and vinaxanthone (Figure 3) [201] were identified as SEMA3A inhibitors, whereby xanthofulvin displayed diminished binding of SEMA3A to NRP1, suggesting a direct interference of the receptor-ligand association [202]. There are no reports on other small molecules targeting the SEMA3/NRP interaction.

## 5. NRP2 Antagonists

Despite the long research and development of NRP1 antagonists, none of the peptidomimetic or the small-molecule antagonists has advanced to clinical studies yet. Although all discovery efforts are focused on NRP1, and no specific NRP2 antagonists are known, there are indications that antagonists targeting NRP2 exclusively are needed. Recently, some attempts were made toward the development of selective NRP2 benzamidine-based antagonists of VEGF-C that exhibited a modest NRP2 potency and provided the basis for further development of NRP2 small molecule antagonists targeting related pathological functions [203].

## 6. Conclusions

In this article, we reviewed the physiological role of NRPs and their association with various pathological conditions. In addition to the discovery of NRP1 as a SARS-CoV-2 entry coreceptor along with ACE2, we have assembled the pathological roles of NRPs and their involvement in clinical diseases, particularly highlighting the participation of NRPs in cancer. NRPs, therefore, represent mature and viable therapeutic targets. Structurally, neuropilin-1 and 2 contain several conserved motifs and domains suitable for a structure-based medicinal chemistry elaboration. In addition, NRPs possess an abundance of endogenous ligands such as SEMA, VEGF, FGF, HGF, PDGF, TGF-β, and miRNAs that facilitate viable ligand-based design approaches and mode of action studies (MOA). The latter is evident from the chemical space of the most successful NRP1 modulators (and chemical probes), which are typically peptidomimetics. However, a handful of other NRP1 small-molecule antagonists have also been reported leaving ample room for future developments. In case the inclined reader would like to learn more about specific NRP-related pathologies, we have compiled the most relevant literature reports in Table 1. For medicinal chemistry, we have provided the most important peptidomimetic and small molecule antagonist structures along with their key receptor interactions in Table 4 and Table 5.

## Figures and Tables

**Table 1:** The main groups and subgroups of the most important endogenous ligands binding to both NRP receptors. x indicates binding to both NRPs.

Endogenous Ligand	Preferences for NRP Binding		Reference
	NRP1	NRP2	
SEMA	SEMA3A SEMA3B SEMA3C SEMA3D SEMA3F	SEMA3B SEMA3C SEMA3D SEMA3F SEMA3G	[2,9]
VEGF	VEGF-A VEGF-A165 VEGF-A189 VEGF-B VEGF-C VEGF-D VEGF-E PIGF	VEGF-A VEGF-A145 VEGF-A165 VEGF-C VEGF-D PIGF	[3,9]
FGF	FGF-1 FGF-2 FGF-4 FGF-7	FGF-2	[18]
HGF	x	x	[19]
PDGF	PDGF-BB PDGF-C PDGF-D	PDGF-BB	[3,20]
TGF-β	TGF-β1	TGF-β1	[21,22]
miRNAs	x	x	[2,23]

**Table 2:** NRP involvement in the entry and/or infectivity of viruses.

Virus	NRP1	NRP2	Reference
HTLV-1	x		[43,45]
EBV	x		[42]
SARS-CoV-2	x		[37,45]
MCMV	x		[46]
EVA71	x		[47]
LUJV		x	[48]
HCMV		x	[49]

## Abbreviations

ACE2, Angiotensin-converting enzyme 2; CendR, C-end rule; CNS, Central nervous system; COVID-19, Coronavirus disease 2019; CUB, C1r/C1s, Uegs, Bmp1, DN; Ddiabetic nephropathy; EBV, Epstein-Barr virus; EVA71, Enterovirus 71; FDA, Food and Drug Administration; FGF, Fibroblast Growth Factor; GLUT-1, Glucose transporter 1; gB, Glycoprotein B; HCMV, Human Cytomegalovirus; HGF, Hepatocyte Growth Factor; HTLV-1, Human T-cell lymphotropic virus type 1; HVV4, Human herpesvirus 4; LPP, Lipoprotein; LUJV, Lujo Virus; MAM, Meprin/A5-protein/PTPmu; MCMV, Murine cytomegalovirus; MOA, Mode of action; NRP(s), Neuropilin(s); NRP1, Neuropilin 1; NRP2, Neuropilin 2; PDGF, Platelet-Derived Growth Factor; PDZ, PSD-95/Dlg/ZO-1; RTK, Receptor tyrosine kinase; SARS-CoV-2, Severe acute respiratory syndrome coronavirus 2; SEMA3, Semaphorin-3; TGF-β, Transform Growth Factor; TM, Transmembrane; VEGF, Vascular endothelial growth factor; VEGFR, Vascular endothelial growth factor receptor.
